# 10-Week Trajectories of Candidate Psychological Processes Differentially Predict Mental Health Gains from Online Dyadic versus Mindfulness Interventions: A Randomized Clinical Trial

**DOI:** 10.3390/jcm13113295

**Published:** 2024-06-03

**Authors:** Malvika Godara, Tania Singer

**Affiliations:** Social Neuroscience Lab, Max Planck Society, 10557 Berlin, Germany; singer@social.mpg.de

**Keywords:** mindfulness, dyad, depression, anxiety, perseverative thinking, acceptance, emotional flexibility, mental training

## Abstract

**Background**: App-based contemplative interventions, such as mindfulness-based interventions, have gained popularity for the promotion of mental health; however, the understanding of underlying intervention-specific mechanisms remains limited, especially related to novel inter-relational dyadic practices. **Methods**: We tested (n = 253) seven putative mechanisms underlying two brief (daily 12-min) online mental interventions: attention-focused mindfulness and socio-emotional partner-based, both supported by weekly online coaching. Weekly self-reports of rumination, worry, psychological flexibility, affective control, social support, acceptance, and mindfulness were obtained over 10 weeks of intervention, and depression, anxiety, and resilience were assessed as pre- and post-intervention outcomes. **Results**: Significant week-to-week reductions in rumination and increases in psychological flexibility were observed in both interventions. Only attention-based practice led to temporal reductions in worry, and only socio-emotional dyadic practice led to temporal increases in affective control. Mediation analyses with slopes of weekly variables as mediators detected no significant indirect effects. However, exploratory moderation analyses revealed that intervention-related reductions in depressive symptomatology and anxiety vulnerability and increases in resilience were predicted by weekly increases in acceptance and affective control in the socio-emotional dyadic group, and by weekly reductions in rumination and worry in the mindfulness group. Limitations of the study include reliance on brief self-report measures, relatively small sample size, and absence of long-term follow-up assessments indicating the need for future well-powered longitudinal studies comparing intervention modalities. **Conclusions**: We present preliminary evidence for practice-specific active ingredients of contemplative interventions, which can be leveraged to enhance their efficiency for mental health.

## 1. Introduction

Depressive and anxiety disorders are consistently ranked among the leading contributors to global health-related burdens [1], underscoring the need for consolidated efforts to intervene and reduce this global burden. Mindfulness-based interventions (MBIs) are an increasingly popular tool to enhance mental well-being. Empirical evidence demonstrates their efficacy for a variety of psychosocial and neurobiological outcomes, including reductions in depression and anxiety, while promoting stress resilience and recovery [2,3,4,5]. However, the understanding of mechanisms of action underlying these interventions remains limited.

Contemporary MBIs trace their origin to the Mindfulness-Based Stress Reduction (MBSR) program [6], and involve a range of practices, such as breathing meditation, self-compassion meditation, and non-judgment and acceptance exercises, making these interventions a mix of various contemplative practices [7,8,9,10,11,12]. A parallel stream of compassion-focused mental interventions, such as the self-compassion program [13] and compassion-focused therapy [14], has also shown efficacy in improving mental health [15,16,17]. Recently, mobile meditation apps incorporating varied practices both from mindfulness-based and compassion-based practices have grown in popularity for mental well-being [18], showing meaningful reductions in psychopathology and improvements in resilience [19,20,21,22].

While mindfulness- and compassion-focused interventions are often treated in a blanket manner to encompass varied practices, conceptual frameworks pushing for a systematic theory-driven investigation of these interventions [5,23,24,25] have posited domain-specific effects of specific practices. This proposition has been confirmed by recent work [5,26,27,28]. For example, in a large-scale 9-month study, the ReSource project [29] employing three distinct intervention modules, it was shown that attention-focused mindfulness practices led to enhanced interoceptive awareness and attention control [30,31], socio-cognitive perspective-taking practices led to improvements in meta-cognitive awareness and Theory of Mind [30,31], and socio-affective practices led to increases in compassion and prosocial behavior and reductions in social stress [31,32,33].

Along with differing practice content, intervention modality is also proposed to be crucial to domain-specific mental health gains [34]. While most MBIs involve solitary practice, recently intersubjective contemplative practices performed with a partner have also emerged, such as inquiry methods [35] and contemplative dialogue [36]. Interpersonal MBIs conducted in group formats have also shown positive outcomes, such as increased social connectedness and reductions in psychopathological symptoms [37,38,39]. Similar benefits, i.e., enhanced social connectedness and disclosure, were observed for a novel partner-based training format, contemplative dyads (e.g., Affect Dyad) that involve a 12 min contemplative dialogue between two individuals focusing on exploring specific questions. For example, in the Affect Dyad practice, the aim is to explore difficult and gratitude-eliciting situations to enhance bodily awareness of emotions and foster acceptance and gratitude [40].

Importantly, the largely heterogeneous investigation of varied MBI practices has yielded a rather mixed understanding of mechanisms underlying these interventions in the mental health context, resulting in a wide range of proposed active ingredients [41,42,43,44]. Some of these mechanisms include use of emotion regulation and coping strategies [45,46,47], mindfulness skills [48,49], psychological flexibility [50], emotional cognitive control [51,52], and worry [53,54]. Conceptual frameworks have proposed that unique active ingredients will underlie content-specific MBI practices [24]. Accordingly, studies have shown that socio-emotional contemplative practices lead to enhanced use of emotion regulation strategies such as acceptance, while attention-focused mindfulness practices are associated with decreased levels of rumination, indicating meditation-specific effects [46,55]. Consequently, a recent study comparing MBSR with compassion cultivation training for mental health showed that MBSR led to improved mental health through enhanced decentering and interoceptive awareness, and compassion training through empathic concern and common humanity [56].

However, a systematic practice-specific understanding of many of the oft-proposed mechanisms of action for mental health remains in nascent stages. State-of-the-art dismantling trials are increasingly used to further mechanistic understanding of MBIs, though studies have produced mixed results [26,27,57,58]. Moreover, mechanisms or treatment response predictors for partner-based practices, such as contemplative dyads, remain largely unexamined given their novelty [59]. Gaining a deeper understanding of practice-specific mechanisms driving mental health improvements will enable us to refine and optimize different contemplative interventions, which in turn might increase their efficiency and potency for mental health and provide new directions for personalized and precision approaches to their application in clinical practice and elsewhere [60].

In the present study, we examined seven different putative mechanisms of two distinct 10-week online mental interventions, attention-focused solitary mindfulness (MB) and socio-emotional dyadic (SE), and evaluated their role in predicting improvements in depression, anxiety, and resilience in a community sample. The MB intervention is geared towards enhancing present-moment awareness and interoceptive body awareness [6], and is performed alone. On the other hand, the SE intervention, Affect Dyad [40], seeks to promote care, compassion, and acceptance of varied emotional states and is performed with a partner. Using data from phase 2 of the CovSocial project [61], we examined whether pre- to post-intervention changes in mental health could be attributed to temporal changes, over 10 weeks of intervention, in the following psychological processes: acceptance, rumination, social support, mindfulness, psychological flexibility, worry, and affective control. In a prior study [59] using the mental health data from phase 2 of the CovSocial project, we were already able to show that MB and SE were equally effective in reducing depressive symptomatology and anxiety vulnerability, compared to a waitlist control group. Moreover, MB led to increases in self-reported levels of stress recovery and SE led to increases in multi-dimensional resilience. Therefore, in the present study, we aimed to examine whether these two interventions may function through unique or shared mechanisms.

The hypotheses for the present study were preregistered on Open Science Framework as part of the Mental Health and Resilience complex of phase 2 of the CovSocial project (osf.io/3nsjc) based on prior research on contemplative practices and their proposed mechanisms for psychopathology and resilience [5,42,43,44,48,62,63,64,65,66]. We hypothesized that reductions in depression will be mediated by changes in acceptance, mindfulness, affective control, psychological flexibility, and social support in SE practice, and by changes in mindfulness, psychological flexibility, and rumination in MB. Further, reductions in anxiety were expected to be mediated by changes in acceptance, mindfulness, and psychological flexibility in SE, and by changes in mindfulness, psychological flexibility, and worry in MB. Lastly, we expected increases in psychological resilience and stress recovery to be mediated by changes in acceptance, mindfulness, affective control, psychological flexibility, and social support in SE, and by changes in mindfulness, psychological flexibility, rumination, and worry in MB practice.

## 2. Materials and Methods

### 2.1. Sample Recruitment

**Figure 1:**
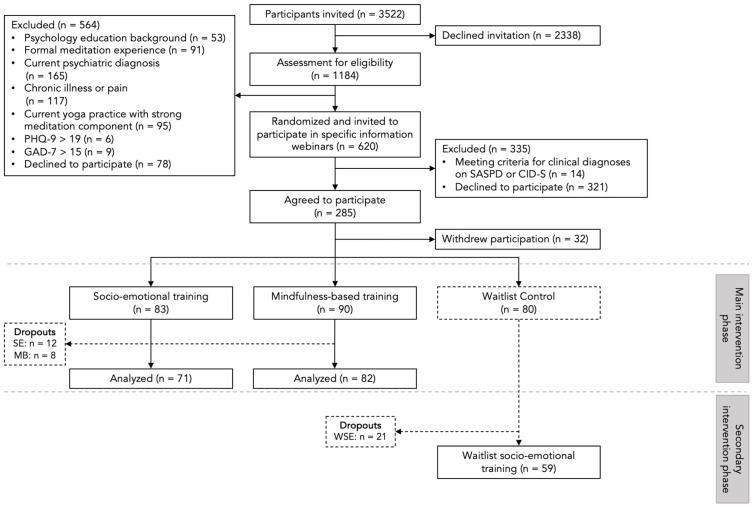
Flow diagram depicting the sample recruitment procedure, exclusion according to pre-registered criteria, and dropouts at each stage of the study. This figure has been adapted from a prior study in the CovSocial project [59]. SE = Socio-emotional intervention, MB = Mindfulness-Based intervention, WSE = Waitlist Socio-emotional intervention.

The present study is embedded in the CovSocial project, which is a multi-phase and multi-domain project. In its first phase, the project examined longitudinal changes in mental health during the COVID-19 pandemic in a sample of Berliners [67]. In a smaller community sample derived from the first phase, we tested two distinct online mental interventions in a randomized controlled trial in phase two of the project (Trial Registration: ClinicalTrials.gov NCT04889508). For the current study, which is part of phase 2, all participants from phase 1 (n = 3522) were first invited to complete a prescreening procedure (Figure 1). Inclusion criteria for participation in phase 2 of the project were the following: 18–65 years old, residing in Berlin, proficiency in the German language, and access to a smartphone capable of supporting the study app. Interested individuals were screened for vulnerability (using the following cut-offs: Patient Health Questionnaire-9 score > 19, Generalized Anxiety Disorder-7 score > 15, or Toronto Alexithymia Scale-20 score > 60), prior meditation practice, chronic illnesses, and current clinically-diagnosed psychiatric disorders, and individuals meeting any of these criteria were excluded from the study. Individuals meeting the selection criteria were then introduced to the study through the “Welcome days” webinars (see Supplementary Information “S1 Mental Training Protocol CovSocial Project Phase 2” for further details). Afterward, participants were randomized into three groups (socio-emotional intervention, mindfulness-based intervention, and waitlist control), and introduced to their specific interventions through “Onboarding sessions”. Participants were then additionally screened for clinical levels of psychopathology using the Standardized Assessment of Severity of Personality Disorder [68] and Composite International Diagnostic Screener [69] through screening calls conducted by trained mindfulness teachers. Upon exclusion and dropouts, the sample at pre-test data collection was as follows: 83 in the SE intervention, 90 in the MB intervention, and 80 in the waitlist control (WC; see Table 1). The study was approved by the Ethics Commission of the Charité –Universitätsmedizin Berlin. Participants provided written informed consent and were compensated 10 euro per hour (see study protocol [61]).

### 2.2. Design and Procedure

**Figure 2:**
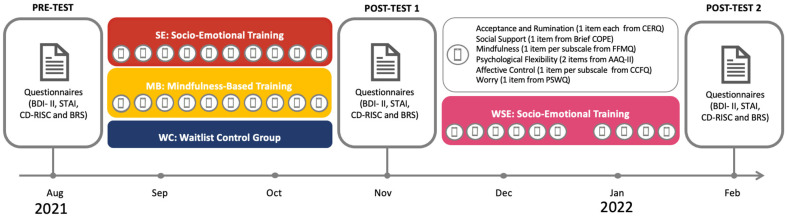
Study design including pre-test, post-test 1, and post-test 2, as well as weekly assessed variables. BDI-II = Beck Depression Inventory-II, STAI = State–Trait Anxiety Inventory, CD-RISC = Connor–Davidson Resilience Scale, BRS = Brief Resilience Scale, CERQ = Cognitive Emotion Regulation Questionnaire, Brief COPE = Brief Coping Orientation to Problems Experienced Inventory, FFMQ = Five Facet Mindfulness Questionnaire, AAQ-II = Acceptance and Action Questionnaire-II, CCFQ = Cognitive Control and Flexibility Questionnaire, and PSWQ = Penn State Worry Questionnaire.

Participants were enrolled and assigned to one of three groups: the SE training, MB training, or WC group. Participants were randomized parallelly in blocks using computer-generated numbers with 1:1:1 allocation. The randomization sequence was generated by a senior researcher and interventions were assigned to participants by the study coordinator. Interventions were administered for 10 weeks using the CovSocial mobile app specifically designed for the study. Over the 10-week intervention period, both SE and MB groups completed weekly assessments of seven putative mechanistic variables using push notifications on the same mobile app (see Figure 2). The WC group later underwent socio-emotional dyadic training in a separate 10-week period, called the waitlist socio-emotional group (WSE), during which they also completed weekly assessments. All participants completed mental health outcome measures before (pre-test) and after (post-test 1) the main intervention phase using the CovSocial app. Participants in the WC/WSE group also completed the mental health outcome measures using the app at an additional third timepoint (post-test 2) after undergoing intervention. Invitation to participate in the study began on 27 May 2021 and data collection for all measures was completed on 31 March 2022.

### 2.3. Measures

#### 2.3.1. Weekly Variables

Acceptance and rumination were assessed using one item each from the Cognitive Emotion Regulation Questionnaire (“I accept difficult situations in my life” and “I am preoccupied with what I think and feel about what I have experienced”; [70,71]). Social support was assessed using one item from the Brief COPE scale (“I’ve been getting help and advice from other people”; [72,73,74]). Worry was assessed with one item from the Penn State Worry Questionnaire (“I worry all the time”; [75,76]). Mindfulness was assessed with five items from the Five Facets of Mindfulness Questionnaire—Short Form [77,78], one for each of the five subscales (“I’m good at finding the words to describe my feelings”, “I pay attention to sensations, such as the wind in my hair or sun on my face”, “I rush through activities without being really attentive to them”, “I tell myself I shouldn’t be thinking the way I’m thinking”, and “I watch my feelings without getting lost in them”). Affective control was assessed using the Cognitive Control and Flexibility Questionnaire [79], one item for each of the two subscales (“My thoughts and emotions interfere with my ability to concentrate” and “I consider difficult situations from multiple viewpoints before responding”). Finally, psychological flexibility was assessed using two items from the Acceptance and Action Questionnaire-II (“Emotions cause problems in my life” and “My painful memories prevent me from having a fulfilling life”; [80,81]). All variables were rated on a 5-point scale ranging from 0 (not at all) to 4 (very much). To minimize respondent burden, avoid a high degree of dropouts due to excessive testing, and streamline data collection, especially given the frequency of weekly assessments, we chose to assess these processes using a limited number of items and to employ a uniform response scale. The selection and adaptation of items for each psychological process were guided by factors such as factor loadings in the respective English and German validation studies, relevance to the study’s objectives, consistency with the intended constructs, and separation from other closely related processes that were being simultaneously assessed in the study.

#### 2.3.2. Mental Health Outcomes

Depression was assessed using the 21-item Beck Depression Inventory-II (BDI-II; [82,83]), and items were rated on a scale from 0 (e.g., “I do not feel sad”) to 4 (e.g., “I am so sad and unhappy that I can’t stand it.”). Trait and state anxiety were assessed using the 40-item State–Trait Anxiety Inventory (STAI; [84,85]). State items assess how people feel in the moment, rated on a scale from “not at all” (1) to “very much so” (4), and trait items assess how people feel generally, rated on a scale from “almost never” (1) to “almost always” (4). Resilience was assessed using the Connor–Davidson Resilience Scale (CD-RISC; [86,87]), which comprises 25 items rated on a scale from 0 (not true at all) to 4 (true nearly all of the time). Stress recovery was measured using the Brief Resilience Scale (BRS; [88,89], which comprises 6 items rated on a scale from 1 (strongly disagree) to 5 (strongly agree). As part of a prior study [59], we were able to show that both SE and MB trainings significantly reduced BDI-II and STAI-Trait scores. In the same study, we showed that SE led to significant increases in CD-RISC scores, and MB led to significant increases in BRS scores. In the present study, therefore, we do not re-discuss the direct intervention-related changes in these mental health outcomes (see Figure S1 for figurative depiction and Table S1 for reliability of the mental health outcome measures) because the present research questions focus on investigating the intervention-specific mechanisms underlying these changes.

### 2.4. Interventions

Before the 10-week intervention period, all participants received a 2.5 h formal introduction to contemplative training, along with two 2.5 h intervention-specific onboarding webinars regarding the theoretical and practical introduction of the interventions (see Supplementary Information “S1 Mental Training Protocol CovSocial Project Phase 2” for a detailed intervention protocol).

#### 2.4.1. Socio-Emotional Dyadic Intervention

Participants in the SE group, and in a later period in the WSE group, engaged in the daily practice of Affect Dyad. It involves a 12.5 min contemplative dialogue with a randomly assigned partner. In the exercise, participants take turns first describing a difficult situation and then a gratitude-eliciting situation of the last 24 h and explore the subjective bodily experience of difficult and gratitude emotions, respectively, during these situations. While one partner is describing the situations and exploring their subjective bodily experience, the other partner listens in an empathic, non-judgmental manner without any verbal or non-verbal communication. Partners were randomly assigned by the app and changed every week.

#### 2.4.2. Attention-Focused Mindfulness Intervention

Participants in the MB group engaged in the daily 12.5 min practice of attention-focused mindfulness exercises. Using app-based audio guides, the primary exercise participants performed was breathing mindfulness. This exercise involves focusing and sustaining attention on breath, and returning to it when the mind wanders. Participants also performed mindfulness on sounds (focusing attention on sounds around them) and open presence mindfulness (focusing attention on sensations in and around them). All exercises were performed individually.

Participants in both interventions were encouraged to engage in the exercises six times a week. Daily practice in both interventions was supported by trained mindfulness teachers through 2 h weekly online coaching sessions to deepen the practice. These sessions were conducted in groups of 14–24 participants, and aimed to enhance understanding of different aspects of respective daily practice and to help integrate it in their life. The topics of coaching sessions in the socio-emotional intervention revolved around social connectedness, empathic non-judgmental listening, interoceptive body awareness, acceptance of difficult emotions or stress, and the cultivation of care and gratitude. The topics of coaching sessions in the mindfulness-based intervention covered the fundamentals of breathing meditation, body awareness, sensory perception, and awareness and dealing with difficult emotions, to foster present-moment attention, interoceptive body awareness, and receptivity towards the self and the body. See Supplementary Information “S1 Mental Training Protocol CovSocial Project Phase 2” for further details.

### 2.5. Statistical Analysis

#### 2.5.1. Power Analysis

Prior to sample recruitment for phase 2 of the CovSocial project, power analysis was conducted utilizing biological measures integral to this phase [61]. A priori effect size and power calculations were derived from prior research validating interventions utilized in the current study [29]. G*Power software (https://www.psychologie.hhu.de/arbeitsgruppen/allgemeine-psychologie-und-arbeitspsychologie/gpower) [90] was employed for the analyses, utilizing analysis of variance with repeated measurements and accounting for interactions between group and intra-group variables. Key parameters included α = 0.05, power = 0.80, three groups, and two measurements, with effect sizes denoted by *r* = 0.39 and *f* = 0.10. These calculations yielded a required sample size of 297 individuals. Consequently, we aimed to recruit approximately 300 individuals, with 100 allocated to each intervention group.

#### 2.5.2. Weekly Variables

Linear mixed models were employed to investigate intervention-related temporal changes in weekly variables on rumination, social support, worry, affective control, and psychological flexibility. For weekly variables that were assessed with more than one item, mean scores were computed across corresponding items. Since weekly variables were not assessed in the WC group during the main intervention phase, the main weekly change analysis focused on SE and MB groups. Fixed effects for intervention, week, and their interaction (intervention x week) were specified with MB as the reference group, and models included random intercepts and slopes. For the WSE group, temporal changes in weekly variables were analyzed with separate random intercept models with a fixed effect of week included in the models. Estimated individual slopes of weekly change were then obtained from these models for the main mediation models. The weekly variables on acceptance and mindfulness that are evaluated as mechanisms of mental health in the current study have also been employed in a prior study [91] from the CovSocial project as mechanisms for intervention-related changes in empathy and compassion. As part of their study, Silveira et al. showed significant increases in acceptance over weeks in MB and significant increases in mindfulness over weeks in all three groups, using the same approach to linear mixed models as in the present study. Therefore, in the present study, we do not discuss the changes in these two processes by themselves in detail but only use the estimated slopes of these weekly variables in the main mediation models and other exploratory analyses.

#### 2.5.3. Mediation Analysis

We tested whether slopes of weekly variables mediated intervention-related changes in mental health outcomes (BDI-II, STAI-T, STAI-S, CD-RISC, and BRS scores) from pre-test to post-test 1. Mediation models were specified with the pre-registered weekly slopes as the mediators, intervention as the dummy-coded predictor (with MB as the reference group), and the post-test 1—pre-test mental health change scores as the dependent measure. All models included sex and age as covariates. Mediation models employed a bootstrapping procedure with 5000 iterations, and bias-corrected confidence intervals are reported.

#### 2.5.4. Exploratory Moderation Analysis

As a final step, we employed linear mixed models to explore whether slopes of weekly variables predicted mental health outcomes and whether this effect was moderated by the intervention received. In the moderation models concerning the main intervention phase, we included separate three-way interaction terms between intervention (SE and MB), time (pre-test and post-test 1), and each of the pre-registered weekly slopes. Separate moderation models were implemented for the WSE group with two-way interaction terms between time (post-test 1 to post-test 2) and the pre-registered weekly slopes.

Mixed effects analyses were conducted using lme4 and multcomp, and mediation models were tested using process, all in R (v.4.1.2; [92]). All reported *p*-values represent the Bonferroni adjusted *p*-values and the significance level was set at *p* < 0.05. Model assumptions were evaluated using visual inspection techniques, such as QQ plots and scatterplots of residuals against fitted values, leading to the detection of no significant violations.

## 3. Results

### 3.1. Weekly Variables

**Figure 3:**
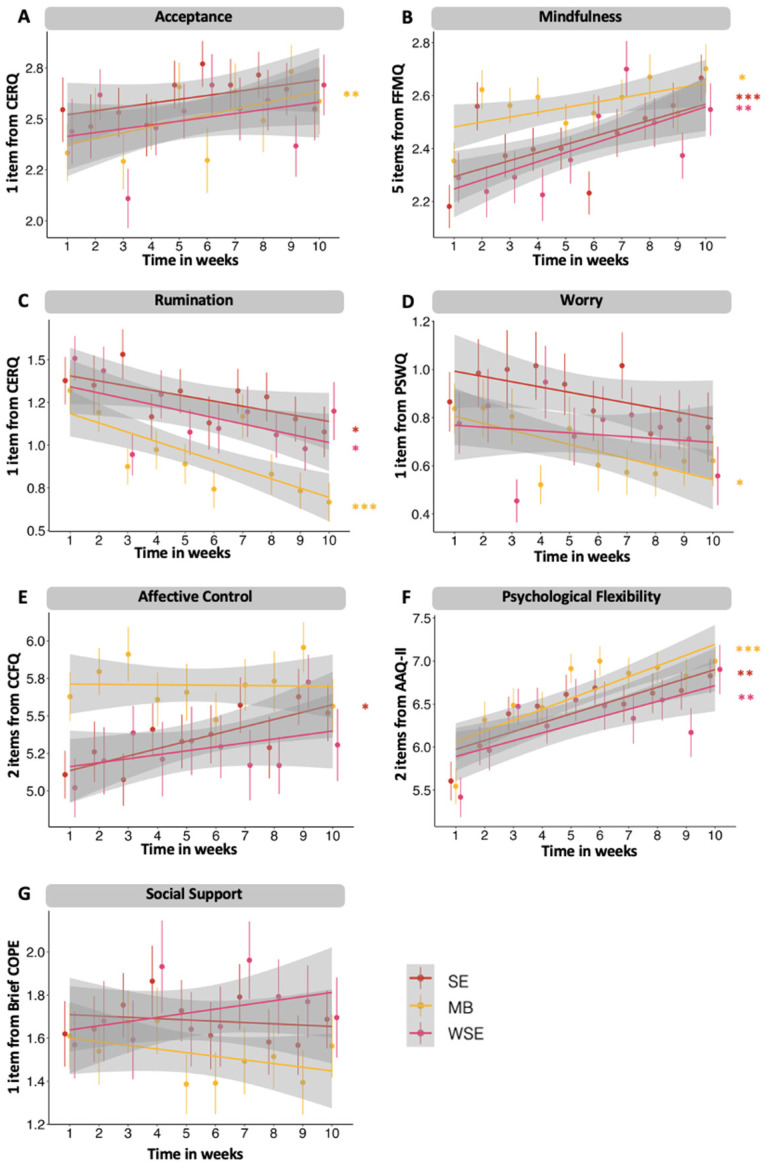
Trajectories of weekly assessed variables over the course of the 10-week intervention period in the socio-emotional (SE) and mindfulness-based (MB) intervention groups. Trajectories are also displayed for the waitlist socio-emotional intervention group (WSE), although they performed their intervention in a later 10-week period. Significance level of * α < 0.05, ** α < 0.05 and *** α < 0.001. CERQ = Cognitive Emotion Regulation Questionnaire, Brief COPE = Brief Coping Orientation to Problems Experienced Inventory, FFMQ = Five Facet Mindfulness Questionnaire, AAQ-II = Acceptance and Action Questionnaire-II, CCFQ = Cognitive Control and Flexibility Questionnaire, and PSWQ = Penn State Worry Questionnaire. Panels A and B have been adapted from a prior study of the CovSocial project [91].

Temporal changes in weekly variables were modeled separately for SE and MB in one model and WSE in a separate model due to different intervention periods. Over the intervention period, both the SE and MB groups demonstrated significant reductions in rumination (*β_SE_* = −0.03, *p* = 0.02; *β_MB_* = −0.05, *p* < 0.001; see Figure 3) and significant increases in psychological flexibility (*β_SE_* = 0.10, *p* < 0.001; *β_MB_* = 0.12, *p* < 0.001). Affective control showed a significant increase only in the SE group (*β_SE_* = 0.04, *p* = 0.03) but not in the MB group (*β_MB_* = 0.003, *p* > 0.05). Conversely, worry significantly decreased only in the MB group (*β_MB_* = −0.03, *p* = 0.01) but not in the SE group (*β_SE_* = −0.02, *p* > 0.05). Social support did not show significant changes in either the SE (*β_SE_* = −0.01, *p* > 0.05) or MB (*β_MB_* = −0.01, *p* > 0.05) groups. There were no significant interactions detected between intervention and week for any variable (*p* > 0.05). In the WSE group, rumination significantly decreased (*β_WSE_* = −0.03, *p* = 0.02) while psychological flexibility significantly increased (*β_WSE_* = 0.08, *p* = 0.003), but there were no significant changes in affective control (*β_WSE_* = 0.03, *p* > 0.05), worry (*β_WSE_* = −0.01, *p* > 0.05), or social support (*β_WSE_* = 0.02, *p* > 0.05). Previously, Silveira et al. [91] reported significant increases in acceptance over weeks only in the MB group (*β_MB_* = 0.04, *p* = 0.002) but not in the SE (*β_SE_* = 0.02, *p* > 0.05) or WSE (*β_WSE_* = 0.02, *p* > 0.05) groups. They also found significant increases in mindfulness over weeks in all three groups (*β_SE_* = 0.03, *p* < 0.001; *β_MB_* = 0.02, *p* = 0.02; *β_WSE_* = 0.03, *p* = 0.002).

### 3.2. Mediation Analysis

Mediation models showed no significant indirect effects of the intervention group on any of the mental health outcomes via any of the preregistered weekly slopes (see Table S2).

### 3.3. Exploratory Moderation Analysis

**Figure 4:**
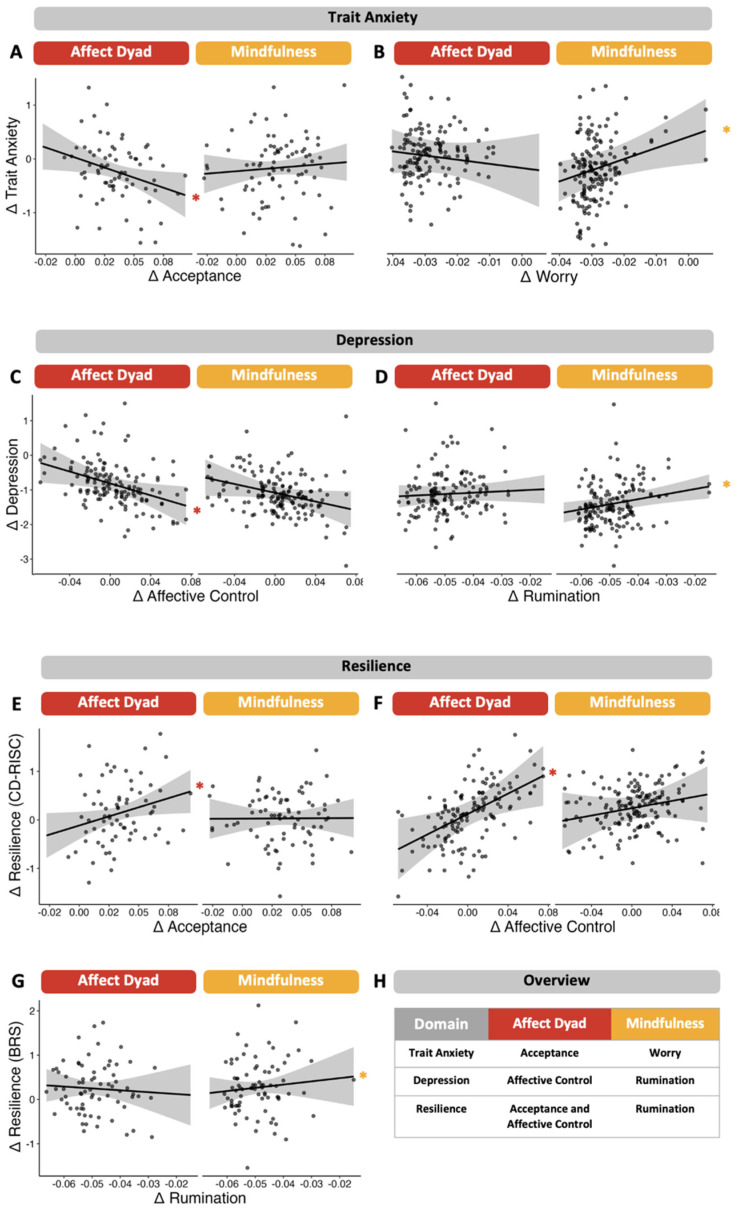
The prediction of changes in mental health outcomes by slopes of weekly assessed variables moderated by intervention received. Panels A and B show associations between changes in trait anxiety and slopes of acceptance and worry, respectively. Panels C and D show associations between changes in depression and slopes of affective control and rumination, respectively. Panels E and F show associations between changes in resilience (Connor–Davidson Resilience Scale; CD-RISC) and slopes of acceptance and affective control, respectively. Panel G shows the association between changes in resilience (Brief Resilience Sale; BRS) and rumination. Panel H summarizes the pattern of findings in a comprehensive overview table. Significance level * α < 0.05.

Moderation analyses revealed that increases in affective control over the intervention period significantly predicted reductions in BDI-II scores in the SE group (*β_SE_* = −6.85, *p* = 0.02), but not in the MB group (*β_MB_* = −4.25, *p* = 0.10; Figure 4). Moreover, increases in acceptance over weeks predicted reductions in STAI-T scores in the SE group (*β_SE_* = −6.02, *p* = 0.009), but not in the MB group (*β_MB_* = 1.03, *p* > 0.05). Furthermore, only in the SE group, increases in CD-RISC scores were significantly associated with increases in acceptance (*β_SE_* = 6.10, *p* = 0.03) and affective control (*β_SE_* = 7.54, *p* = 0.02), but not in MB (*p*s > 0.05). Contrastingly, only in the MB group, decreases in rumination over 10 weeks significantly predicted reductions in BDI-II scores (*β_MB_* = 7.10, *p* = 0.01) and increases in BRS scores (*β_MB_* = −9.75, *p* < 0.001), but not in the SE group (*β_BDI-II_* = −1.14, *p* > 0.05 and *β_BRS_* = 7.10, *p* = 0.01). Moreover, in the MB group, decreases in worry predicted reductions in STAI-T scores (*β_MB_* = 6.93, *p* = 0.009), but not in the SE group (*β_SE_* = −1.75, *p* > 0.05). The type of intervention received did not significantly moderate relationships between other weekly variables (psychological flexibility, mindfulness and social support) and any of the mental health outcomes (all *p*s > 0.05). Furthermore, there were no significant moderation effects of the intervention for associations between any of the weekly variables and STAI-S scores (all *p*s > 0.05). Lastly, in the WSE group, we did not find any significant interactions between time and slopes of weekly variables for mental health outcomes (see Table S3).

## 4. Discussion

In the present study, embedded in the second phase of the CovSocial project [61], we evaluated the potentially common and unique mechanisms underlying two distinct mental trainings, socio-emotional dyadic (SE) and attention-focused mindfulness (MB), in the context of training-related changes in mental health. We tracked seven putatively-relevant psychological processes over 10 weeks of interventions through weekly assessments using a mobile app and examined their association with intervention-related changes in depression, anxiety, and resilience. We found that over 10 weeks, SE practice led to temporal increases in affective control, while MB led to temporal decreases in worry. Both practices led to reduced rumination and increased psychological flexibility over the weeks. These findings support the efficacy of online brief contemplative trainings for temporal changes in perseverative thinking and flexibility processes. In a prior study from the project [91], we already showed that acceptance increased significantly in the MB practice and mindfulness increased in both interventions.

Both interventions led to weekly reductions in rumination, a repetitive negative thinking pattern associated with depressive and anxiety disorders [93,94]. However, only MB intervention led to significant temporal reductions in worry, which involves perseverative thinking about future threats [95,96]. Previous studies have shown the effectiveness of MBIs in reducing perseverative thinking [54,97], and prior studies [30,98] have demonstrated that attention-focused meditation reduces past- and future-related thoughts while socio-emotional practices increase positive other-related thoughts. Our findings extend this prior work by showing that different practices lead to temporal reductions in specific components of perseverative thinking.

Both interventions were also successful in increasing psychological flexibility, but only SE practice showed training-related increases in affective control. This implies that although both practices led to reductions in experiential avoidance and increases in openness [50], only SE practice led to greater control over emotions and the ability to find multiple alternatives and solutions to life’s difficult situations [99,100]. This could be related to the nature of Affect Dyad, wherein the ability to shift between contemplation and acceptance of difficult and positive emotions is trained. Since little research has directly examined the impact of MBIs on these aspects underlying psychopathology [51,101,102,103], our findings add to the current knowledge. Lastly, we found no significant changes in social support, which could be attributed to the measure of social support employed in the present study assessing instrumental support [72].

In the present study, mediation analyses did not yield significant findings, indicating insufficient evidence to make causal claims, which is perhaps a consequence of the small sample size in the present study. While these initial null mediation findings preclude us from making mechanistic claims, the results from the exploratory moderation analyses allow us to speak about treatment response predictors for specific interventions that explain when a certain intervention has beneficial effects on mental health. Moderation analyses revealed that in the SE group, increases in acceptance were associated with intervention-related reductions in anxiety vulnerability, and improvements in affective control were predictive of reduced depression symptom severity. Further, increases in both acceptance and affective control predicted increased levels of multi-dimensional resilience. On the other hand, in the MB group, weekly reductions in worry were associated with a decline in anxiety vulnerability. Moreover, decreases in rumination predicted reductions in depressive symptomatology and increases in stress recovery.

As such, we were able to delineate specific response predictors associated with these two distinct interventions for reducing depression symptomatology and anxiety vulnerability and increasing resilience. Conceptual models of psychopathology and prominent perspectives on MBIs have consistently proposed certain key underlying psychological processes, such as emotion regulation and mindfulness skills [5,24,42,43,44,45,48,50,63,64,65,66]. We extend these findings to the novel partner-based socio-emotional intervention and show that these two interventions seem to be associated with mental health gains in distinct ways. In the MB intervention, reductions in psychopathology are associated with the disruption of perseverative thought patterns and the creation of equanimity [104]. Contrastingly, in the SE practice, mental health improvements are associated with fostering acceptance and affective control as it enhances tolerance of varied emotional states, including challenging emotions experienced in daily life. These findings are also in line with earlier observations that only the SE, and not the MB, intervention increases positive interpretation bias, which in turn mediates decreases in depression and trait anxiety [59]. Even though both interventions also enhance the broader constructs of mindfulness and psychological flexibility, mental health response seems to be predicted by the specific sub-components of these broader constructs, such as affective control in the case of SE, which further provides nuance to our understanding of contemplative interventions.

Given that these two practices influence unique active psychological processes, application of these interventions could benefit from a more personalized approach. For example, use of attention-based MBIs could be more appropriate for individuals displaying elevated levels of worry, while socio-emotional dyadic interventions may be more beneficial for individuals reporting lower levels of affective control. In line with this view, a recent study from the CovSocial project found socio-emotional dyadic intervention to be particularly efficient in boosting affiliative and social capacities such as social connectedness in relations to others which led to specific declines in loneliness [105]. In addition, our findings underscore the importance of testing these interventions in populations displaying clinically relevant levels of active ingredients, ensuring their applicability and efficacy in real-world settings. Moreover, adopting a personalized approach based on these active ingredients could significantly enhance clinical outcomes and treatment effectiveness. For example, in a recent study testing personalization of interventions in the CovSocial project, we could show that those with greater plasticity could benefit more from brief app-based interventions [106]. As such, intervention applications driven by targeting specific active ingredients could lead to more potent improvements in mental health outcomes. Therefore, these two types of app-based low-dose mental trainings used in the current study, especially the novel and understudied socio-emotional dyadic intervention, could be tested in varied populations to optimize them for precise application.

### Limitations

First, the current community sample, though rather heterogenous, had an overrepresentation of females and low representation of migration background diversity, which deviates from the Berlin population [107]. Furthermore, in the control group that later received the SE intervention, no significant moderation effects could be detected. However, this could be attributed to the smaller sample size owing to higher attrition rates in this group as compared to the other two main groups. Moreover, although the present study represents an important advance in understanding practice-specific mechanisms, we only used single items from self-report scales to assess the active ingredients on a weekly basis during the intervention program. Therefore, future studies with better representative samples, more well-powered designs, and using more extensive questionnaires and objective tasks or markers to assess mechanisms may provide further insights. Moreover, the SE intervention employed in the current study does not allow for differentiation between the effects of the socio-affective content and the intersubjective modality of this partner-based practice, which will have to be disentangled by future studies. One limitation of our study is the use of a uniform 5-point rating scale (0 = not at all, 4 = very much) across all weekly items that were in fact derived from various validated questionnaires. While this decision was made to simplify the participant experience and reduce cognitive load given the frequency of weekly assessments as well as provide comparability across all weekly assessed processes in our study, it may affect the direct comparability of our findings with those from studies using the original rating scales. Different rating scales tailored to specific constructs might capture nuances more accurately. Future research should consider these potential discrepancies and aim to balance participant burden with the need for scale-specific precision in measurement. Finally, a limitation of our study concerns the timeframe for post-intervention assessments, with assessments taking place immediately after the end of the intervention period. As a result, the detected effects should be considered short term and future research should include longer follow-up periods to assess the dynamics and sustainability of these intervention effects over time.

## 5. Conclusions

The present study investigated the psychological processes associated with two distinct online mental interventions, mindfulness-based and the Affect Dyad, in the context of intervention-related changes in mental health. The interventions uniquely influenced temporal changes in the weekly assessed psychological processes. We found that reductions in depressive symptomatology and anxiety and increases in resilience and stress recovery, observed after both interventions, were predicted by week-to-week increases in acceptance and affective control in the Affect Dyad group and by temporal reductions in rumination and worry in the mindfulness-based intervention. The present findings advance our understanding of practice-specific active ingredients, paving the way for potential personalized approaches to app-delivered interventions.

## Figures and Tables

**Table 1:** An overview of the sample descriptives (n = 253). This table has been adapted from a prior study in the CovSocial project [59].

Characteristic	Socio-Emotional Intervention	Mindfulness-Based Intervention	Waitlist Control Group
N	83	90	80
Age in years, mean (SD)	43.14 (11.80)	44.14 (11.44)	45.86 (11.15)
Females, N (%)	65 (78.3%)	64 (71.1%)	62 (77.5%)
Background of migration to current country of residence, N (%)	4 (4.8%)	10 (11.1%)	3 (3.8%)
Years of education, mean (SD)	18.49 (3.97)	17.06 (3.52)	18.41 (3.21)
Married or cohabiting, N (%)	27 (32.5%)	32 (35.6%)	32 (40%)
Lifetime prevalence of psychiatric disorder	17 (21.0%)	16 (17.8%)	18 (22.5%)
Income > Berlin average monthly net (€2175)	52 (62.7%)	61 (67.8%)	56 (70.9%)
Employed full-time, N (%)	42 (50.6%)	57 (63.3%)	46 (57.5%)

Note: The average monthly net household income for Berlin is EUR 2175 as reported by Office of Statistics (Amt für Statistik) Berlin-Brandenburg, 2019.

## Data Availability

Data will be made available upon request.

## Supplementary Materials

The following supporting information can be downloaded at: https://www.mdpi.com/article/10.3390/jcm13113295/s1, Figure S1: Effects of socio-emotional and mindfulness-based interventions on primary outcomes; S1 Mental Training Protocol CovSocial Project Phase 2; Table S1: Cronbach’s alpha for mental health outcome measures at each timepoint; Table S2: Indirect effects of intervention on changes in mental health outcomes (post-test 1–pre-test) via weekly variable slopes; Table S3: Regression estimates of weekly variable slopes on outcome measures in the WSE group.
