# Supplementary material for: 10-Week Trajectories of Candidate Psychological Processes Differentially Predict Mental Health Gains from Online Dyadic versus Mindfulness Interventions: A Randomized Clinical Trial
Source: J Clin Med. 2024 Jun 3;13(11):3295. doi: 10.3390/jcm13113295 (PMC11172466; doi:10.3390/jcm13113295)

Figure S1. Effects of socio-emotional and mindfulness-based interventions on primary outcomes. Line graphs depict the mean score of the primary outcomes at each timepoint for each intervention group. Bar graphs show the difference scores from pre-test to post-test 1 for SE, MB and WC groups for all the primary outcomes. Difference scores are also shown for the WC from post-test 1 to post-test 2 after they undergo socio-emotional intervention. Error bars represent standard error. BDI-II = Beck Depression Inventory-II, STAI-T = State-Trait Anxiety Inventory-Trait, STAI-S = State-Trait Anxiety Inventory-State, CD-RISC = Connor-Davidson Resilience Scale, BRS = Brief Resilience Scale.

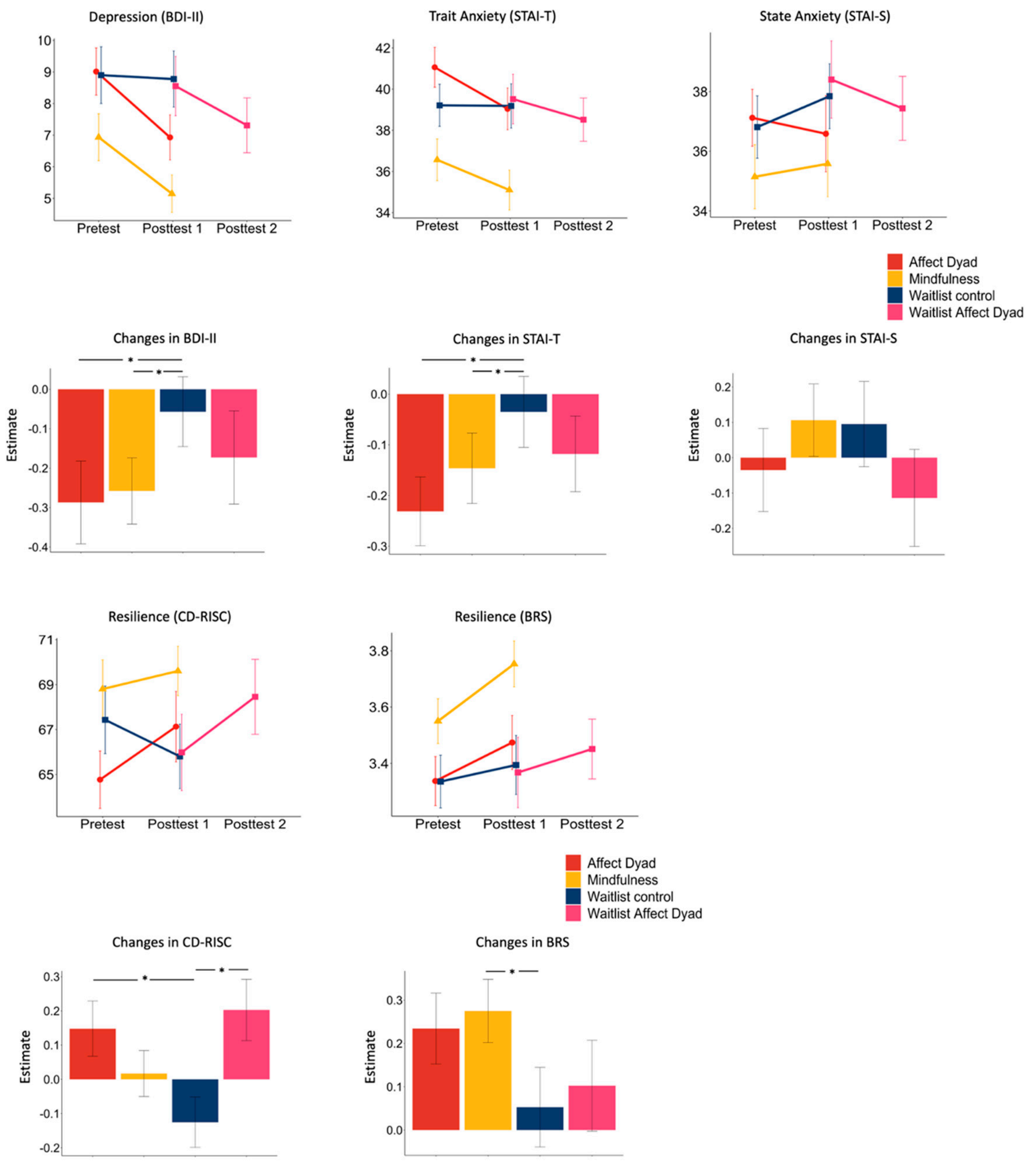

Supplement: Supplementary file 1 [file jcm-13-03295-s001.zip › Figure S1.pdf]
