# Supplementary material for: 10-Week Trajectories of Candidate Psychological Processes Differentially Predict Mental Health Gains from Online Dyadic versus Mindfulness Interventions: A Randomized Clinical Trial
Source: J Clin Med. 2024 Jun 3;13(11):3295. doi: 10.3390/jcm13113295 (PMC11172466; doi:10.3390/jcm13113295)
Supplement: Supplementary file 1 [file jcm-13-03295-s001.zip › Table S1.pdf]

**Table S1**. Cronbach's alpha values are provided for Beck Depression Inventory-II (BDI-II), STAI-T (State Trait Anxiety Inventory-Trait), STAI-S (State Trait Anxiety Inventory-State), Connor Davidson Resilience Scale (CD-RISC), and Brief Resilience Scale (BRS).

| | **Pre-test (n = 253)** | **Post-test 1 (n = 223)** | **Post-test 2 (n =58)** |
|---|---|---|---|
| **Depression (BDI-II)** | 0.90 | 0.89 | 0.89 |
| **Trait Anxiety (STAI-T)** | 0.92 | 0.92 | 0.89 |
| **State Anxiety (STAI-S)** | 0.92 | 0.93 | 0.88 |
| **Resilience (CD-RISC)** | 0.90 | 0.89 | 0.90 |
| **Resilience (BRS)** | 0.86 | 0.87 | 0.83 |
