# Supplementary material for: 10-Week Trajectories of Candidate Psychological Processes Differentially Predict Mental Health Gains from Online Dyadic versus Mindfulness Interventions: A Randomized Clinical Trial
Source: J Clin Med. 2024 Jun 3;13(11):3295. doi: 10.3390/jcm13113295 (PMC11172466; doi:10.3390/jcm13113295)
Supplement: Supplementary file 1 [file jcm-13-03295-s001.zip › Table S2.pdf]

Table S2. Indirect effects of intervention on changes in mental health outcomes (post-test 1 – pre-test) via weekly variable slopes. BDI-II = Beck Depression Inventory-II, STAI-T = State-Trait Anxiety Inventory-Trait, STAI-S = State-Trait Anxiety Inventory-State, CD-RISC = Connor-Davidson Resilience Scale, BRS = Brief Resilience Scale.

	**Weekly variable slope**	**Indirect Effect [CI_{LL}, CI_{UL}]**
Depression (BDI-II)	Acceptance	-0.0005 [-0.02, 0.03]
	Affective Control	-0.0194 [-0.07, 0.04]
	Psychological Flexibility	-0.0007 [-0.02, 0.03]
	Mindfulness	-0.0012 [-0.04, 0.04]
	Social Support	-0.0007 [-0.03, 0.02]
	Rumination	-0.0193 [-0.07, 0.01]
Trait Anxiety (STAI-T)	Acceptance	-0.0058 [-0.05, 0.03]
	Psychological Flexibility	-0.0004 [-0.02, 0.02]
	Mindfulness	-0.0010 [-0.04, 0.03]
	Worry	-0.0201 [-0.07, 0.03]
State Anxiety (STAI-S)	Acceptance	-0.0032 [-0.04, 0.03]
	Psychological Flexibility	-0.0001 [-0.03, 0.03]
	Mindfulness	-0.0015 [-0.05, 0.06]
	Worry	-0.0307 [-0.13, 0.04]
Resilience (CD-RISC)	Acceptance	0.0043 [-0.04, 0.04]
	Affective Control	0.0074 [-0.02, 0.05]
	Psychological Flexibility	-0.0030 [-0.03, 0.02]
	Mindfulness	0.0032 [-0.02, 0.03]
	Social Support	-0.0004 [-0.02, 0.02]
	Rumination	0.0089 [-0.02, 0.04]
	Worry	0.0027 [-0.03, 0.04]
Resilience (BRS)	Acceptance	0.0010 [-0.02, 0.02]
	Affective Control	0.0094 [-0.03, 0.05]
	Psychological Flexibility	0.0070 [-0.04, 0.05]
	Mindfulness	0.0002 [-0.02, 0.02]
	Social Support	0.0007 [-0.02, 0.02]
	Rumination	0.0114 [-0.02, 0.05]
	Worry	0.0070 [-0.07, 0.07]
