# Supplementary material for: 10-Week Trajectories of Candidate Psychological Processes Differentially Predict Mental Health Gains from Online Dyadic versus Mindfulness Interventions: A Randomized Clinical Trial
Source: J Clin Med. 2024 Jun 3;13(11):3295. doi: 10.3390/jcm13113295 (PMC11172466; doi:10.3390/jcm13113295)
Supplement: Supplementary file 1 [file jcm-13-03295-s001.zip › Table S3.pdf]

Table S3. Regression estimates of weekly variable slopes on outcome measures in the WSE group (all *p*s > .05). BDI-II = Beck Depression Inventory-II, STAI-T = State-Trait Anxiety Inventory-Trait, STAI-S = State-Trait Anxiety Inventory-State, CD-RISC = Connor-Davidson Resilience Scale, BRS = Brief Resilience Scale.

	**Weekly variable slope**	**Estimate (*standard error*)**
Depression (BDI-II)	Acceptance	-1.01 (2.03)
	Affective Control	0.31 (2.08)
	Psychological Flexibility	-0.08 (.48)
	Mindfulness	1.45 (1.72)
	Social Support	-0.01 (1.91)
	Rumination	1.36 (3.43)
Trait Anxiety (STAI-T)	Acceptance	-1.79 (1.70)
	Psychological Flexibility	-0.05 (0.39)
	Mindfulness	-1.45 (1.24)
	Worry	-0.05 (0.04)
State Anxiety (STAI-S)	Acceptance	-1.17 (1.70)
	Psychological Flexibility	-0.35 (0.39)
	Mindfulness	0.55 (1.31)
	Worry	-0.08 (0.04)
Resilience (CD-RISC)	Acceptance	-0.18 (2.05)
	Affective Control	-0.17 (2.14)
	Psychological Flexibility	0.06 (0.49)
	Mindfulness	3.01 (1.73)
	Social Support	0.86 (1.92)
	Rumination	-0.41 (3.11)
	Worry	0.04 (0.05)
Resilience (BRS)	Acceptance	3.12 (2.03)
	Affective Control	-2.66 (2.10)
	Psychological Flexibility	0.52 (0.48)
	Mindfulness	1.39 (1.71)
	Social Support	1.84 (1.88)
	Rumination	2.22 (3.29)
	Worry	0.05 (0.05)
